# The MARINA Risk Assessment Strategy: A Flexible Strategy for Efficient Information Collection and Risk Assessment of Nanomaterials

**DOI:** 10.3390/ijerph121214961

**Published:** 2015-11-27

**Authors:** Peter M. J. Bos, Stefania Gottardo, Janeck J. Scott-Fordsmand, Martie van Tongeren, Elena Semenzin, Teresa F. Fernandes, Danail Hristozov, Kerstin Hund-Rinke, Neil Hunt, Muhammad-Adeel Irfan, Robert Landsiedel, Willie J. G. M. Peijnenburg, Araceli Sánchez Jiménez, Petra C. E. van Kesteren, Agnes G. Oomen

**Affiliations:** 1 National Institute for Public Health and the Environment (RIVM), PO Box 1, Bilthoven 3720 BA, The Netherlands; Willie.Peijnenburg@rivm.nl or peijnenburg@cml.leidenuniv.nl (W.J.G.M.P.); Petra.van.Kesteren@rivm.nl (P.C.E.K.); Agnes.Oomen@rivm.nl (A.G.O.); 2 European Commission, Joint Research Centre, Via E. Fermi 2749, Ispra (VA) 21027, Italy; Stefania.GOTTARDO@ec.europa.eu; 3 Department of Bioscience, Aarhus University, Vejlsøvej 25, PO Box 314, Silkeborg 8600, Denmark; jsf@bios.au.dk; 4 Institute of Occupational Medicine, Centre for Human Exposure Science (CHES), Research Avenue North, Riccarton, Edinburgh EH14 4AP, UK; Martie.VanTongeren@iom-world.org (M.T.); Araceli.Sanchez@iom-world.org (A.S.J.); 5 Department of Environmental Sciences, Informatics and Statistics, University Ca’ Foscari of Venice, c/o VEGApark, Via delle Industrie 21/8, Marghera (VE) 30175, Italy; Semenzin@unive.it (E.S.); Danail.Hristozov@unive.it (D.H.); 6 School of Life Sciences, Heriot-Watt University, Edinburgh EH14 4AS, UK; T.Fernandes@hw.ac.uk; 7 Fraunhofer Institute for Molecular Biology and Applied Ecology, Auf dem Aberg 1, Schmallenberg 57392, Germany; Kerstin.Hund-Rinke@ime.fraunhofer.de; 8 The REACH Centre, Gordon Manley Building, Lancaster Environment Centre, Lancaster University, Lancaster LA1 4YQ, UK; N.Hunt@thereachcentre.com; 9 Experimental Toxicology and Ecology, BASF SE, GB/TB-Z470, Ludwigshafen 67056, Germany; muhammad-adeel.irfan@basf.com (M.-A.I.); robert.landsiedel@basf.com (R.L.); 10 Centre for Environmental Sciences, University Leiden, PO Box 9518, 2300 RA Leiden, The Netherlands

**Keywords:** nanomaterials, risk assessment strategy, exposure-driven, problem framing

## Abstract

An engineered nanomaterial (ENM) may actually consist of a population of primary particles, aggregates and agglomerates of various sizes. Furthermore, their physico-chemical characteristics may change during the various life-cycle stages. It will probably not be feasible to test all varieties of all ENMs for possible health and environmental risks. There is therefore a need to further develop the approaches for risk assessment of ENMs. Within the EU FP7 project Managing Risks of Nanoparticles (MARINA) a two-phase risk assessment strategy has been developed. In Phase 1 (Problem framing) a base set of information is considered, relevant exposure scenarios (RESs) are identified and the scope for Phase 2 (Risk assessment) is established. The relevance of an RES is indicated by information on exposure, fate/kinetics and/or hazard; these three domains are included as separate pillars that contain specific tools. Phase 2 consists of an iterative process of risk characterization, identification of data needs and integrated collection and evaluation of data on the three domains, until sufficient information is obtained to conclude on possible risks in a RES. Only data are generated that are considered to be needed for the purpose of risk assessment. A fourth pillar, risk characterization, is defined and it contains risk assessment tools. This strategy describes a flexible and efficient approach for data collection and risk assessment which is essential to ensure safety of ENMs. Further developments are needed to provide guidance and make the MARINA Risk Assessment Strategy operational. Case studies will be needed to refine the strategy.

## 1. Introduction

Engineered nanomaterials (ENM) are being rapidly developed with many variations of size, shape, structure, and surface modifications, for example, and to such an extent that risk assessment of these ENMs is a challenging process. Not in the least this is because ENMs of a particular chemical composition may actually consist of a population of primary particles, aggregates and agglomerates of e.g., various sizes, and different surface coatings with characteristics that may change over time [1]. Their physico-chemical characteristics may influence their general environmental/ambient fate, the exposure scenarios, the subsequent fate in ecosystems and kinetics within an organism (ecological or human), and on the subsequent extent and type of biological effects, hence on potential health and environmental risks [2,3,4,5,6].

This poses specific demands upon the risk assessment process for ENMs and the information needed. Adequate and sufficient information is required to ensure the safety of ENMs as such and their use in products. However, requiring full testing of slightly different ENMs may lead to unreasonable costs and at the same time may challenge the intention to reduce animal testing. Taking up all these issues requires a risk assessment strategy that considers the impact on human health and the environment of the variation in properties of an ENM during all the relevant life cycle stages, from production to formulation, (consumer) use, recycling and, finally, waste. Such a strategy should provide guidance for an efficient data generation and collection but at the same time be sufficient to ensure a reliable assessment of human health or environmental risks. Since it will simply not be feasible to test all ENMs in all their varieties, an important objective of such an approach is to enable grouping and read-across approaches which will allow sharing of data or information between ENMs and/or bulk-form(s), either to target further testing or to use existing information to fill data gaps. Taking up the gauntlet to meet these needs and challenges for ENMs, the present paper describes a novel risk assessment strategy that has been developed outside regulatory contexts. The strategy aims to identify the best way to perform the risk assessment of ENMs with minimizing data generation. The presented risk assessment strategy is expected to serve the purpose of different users such as industry during the design-phase of an ENM or by regulators within a regulatory framework.

### 1.1. Present Initiatives and Views

In recent years, several initiatives for the development of new risk assessment strategies have been taken, most of them however not specifically aiming at ENMs. A major initiative for chemicals in general is the Tox21 program, a federal collaboration in the USA aiming to improve toxicity assessment and to provide a framework for next generation risk assessment. Tox21 focuses on the development and use of high-throughput *in vitro* methods, mechanism-based biology, broad coverage of chemicals and life stages and minimal use of animals for testing [7,8,9]. Another view on future risk assessment was provided by the EU Scientific Committees [10], focussing on organic chemicals. Distinctively different roadmaps are foreseen for environmental and human health risk assessments. As to human risk assessment, the EU Scientific Committees see a likely paradigm shift from a hazard-driven risk assessment to an exposure-driven approach. In their view, there is a need/trend to change the basis of human health risk assessment from the one based on standard tests to one that is centred on mode of action, bringing about a need for improved and validated databases. There is also a need to develop/facilitate development of (Q)SAR and read-across, (analytical) improvements in the identification and characterization (see also Tantra *et al.* [11]) and development of tools that can increase the ecological realism of exposure and effect assessment. Regarding environmental risks, the EU Scientific Committees conclude that current approaches lack sufficient realism. Among others, it is proposed to develop new models for realistic scenarios, also taking e.g., environmental factors interacting with ENMs into account, and to seek for agreement on relevant test species and endpoints. Opinions on the risk assessment of ENMs specifically were published by SCENIHR [12] and OECD [13]. Both concluded that the current risk assessment approach is in principle applicable to ENMs and that the current methodologies are able to identify the hazards, with the caveat that it was recommended to pay attention to the physico-chemical properties of ENMs and to changes in these properties in relation to hazard and risk assessment of ENMs. Further, attention should be paid to sample preparation [14], dose metrics, inhalation as important exposure route, assessment on a case-by-case basis and tiered testing strategies.

In line with the above opinions, several EU projects have proposed improvements to the risk assessment methodologies of ENMs. The EU funded project ITS-NANO running from 2012 to 2013 aimed to generate a research strategy in the future that could lead to an Intelligent Testing Strategy for ENMs. The project proposed a framework for an intelligent testing approach for risk assessment of ENMs and a prioritization of research needs [15]. ITS-NANO focused on methodological aspects that still require further research and development. More recently, discussions in the NanoSafety Cluster Group (WG) 10 resulted in the publication of a vision for concern-driven integrated approaches to testing and assessment (IATA) of ENMs [4]. A tiered approach was presented in which material properties, exposure, kinetics and hazard data are integrated for the purpose of accelerating the risk assessment process and reducing testing costs and animal use and ensure safety of ENMs and their application. Further, the EU funded project NANoREG (a common European approach to the regulatory testing of ENMs (http://nanoreg.eu)) aims to provide legislators with a proposal for an overarching framework including a set of tools for risk assessment, decision making instruments and new testing strategies in close collaboration with industry. For this purpose, hazard and exposure data are gathered for a number of selected ENMs. Recently ECETOC published a decision-making framework for the testing and grouping of nanomaterials, which uses exposure and “functionality” (system-dependent material properties) of nanomaterials as decision criteria rather than relying on intrinsic material properties alone [3].

### 1.2. Preconditions for A Risk Assessment Strategy for ENMs

A risk assessment strategy for ENMs should be flexible and efficient and also ensure the safety of ENMs for human health and the environment. Flexibility is required to be able to address different assessment goals depending on the user and her/his needs, considering as starting point all data already available and subsequently selecting the most appropriate tools to fill the identified data gaps. Efficiency is needed to primarily collect the data and information that are needed for the risk assessment of an ENM based on the user’s goals (*i.e.*, targeted testing for the purpose of e.g., the ENM design-phase, a risk assessment within a particular regulatory framework, *etc.*) instead of fulfilling predefined data requirements. The strategy will enable to choose the best option forward. Efficiency is also achieved through enabling the possibility to apply information from a source material to a target material (*i.e.*, read-across) or sharing information or highlighting information needs or read-across options early in the risk assessment process. With flexibility and efficiency as important drivers, an optimal balance can be assured between compiling the best quality data and useful information and the effort required to generate these data and information.

Before embarking on the risk assessment the scope of the exercise should be clearly defined (e.g., what ENM or group of ENMs, all or selected life-cycles, human health and/or the environment, exposure routes, health outcomes, *etc.*). The risk assessment strategy shall include the following prerequisites:

- Identification of all relevant exposure scenarios during the life cycle of an ENM. This may for instance relate to scenarios with a specific exposure route and the highest potential of exposure or to a specific life-cycle stage of concern, depending on the assessment goals of the user. It is noted that relevance of an exposure scenario can be indicated by information on exposure, fate/kinetics or hazard or any combination of these domains. These initial considerations shall be based on all available data; it is acknowledged that these data will be limited for an ENM under development.
- Definition of a minimum base set of obligatory information, in order to be able to identify relevant exposure scenarios.
- Verification whether the exposure scenarios need further consideration, *i.e.*, that it cannot be ruled out that a human health and/or environmental risk may be present.
- If risk cannot be ruled out, the type of information that best serves the risk assessment process needs to be determined and guidance should be provided how to obtain the required information. Data gaps should be addressed in a stepwise process starting with relatively simple/inexpensive methods or tools and moving to more sophisticated tools, as needed further down the risk assessment process. Only data should be generated that benefits the risk assessment process.

Starting from these prerequisites, the concept for a risk assessment strategy has been developed within the FP7 project Managing Risks of Nanoparticles (MARINA; grant agreement No. 263215). The present paper describes the blueprint of this strategy, which will be operationalized in other papers [16,17] or later project reports. First, the main outline will be sketched, providing an overview of the framework and the positioning of its main elements and describing the basic process. The main elements are described in more detail in the subsequent sections.

## 2. The MARINA Risk Assessment Strategy

### 2.1. Outline

**Figure 1:**
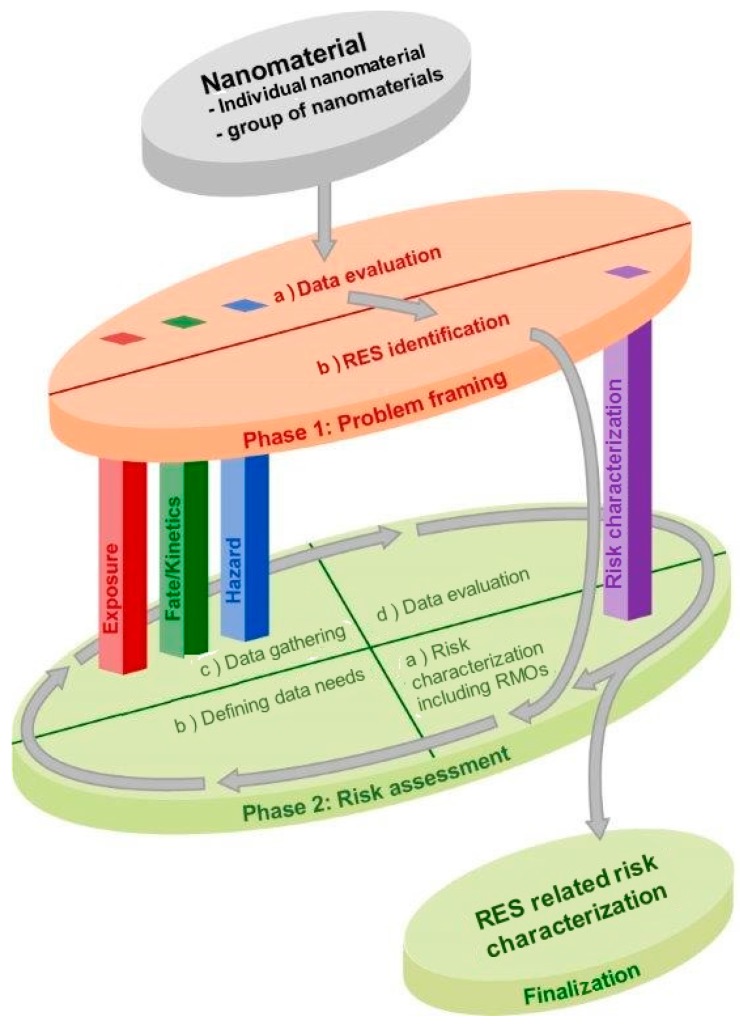
Schematic overview of the MARINA Risk Assessment Strategy, consisting of: (1) an overarching “Phase 1: Problem framing” (orange disc); (2) the iterative “Phase 2: Risk assessment” (green discs: cyclic evaluation process and a finalization step); (3) the three information-gathering pillars: Exposure (red), Fate/Kinetics (green) and Hazard (blue) and (4) the Risk characterization pillar (purple). Phase 1 consists of two steps: (a) Data evaluation; and (b) identification of Relevant Exposure Scenarios (RESs). The iterative evaluation process of Phase 2 consists of four steps: (a) Risk characterization including risk management options (RMOs); (b) Defining data needs; (c) Data gathering and (d) Data evaluation. (See text for further explanation).

In brief, the MARINA Risk Assessment Strategy is composed of two subsequent phases and four pillars which are closely linked (Figure 1). The two phases include “Phase 1: Problem framing” and “Phase 2: Risk assessment” consisting of a refined assessment starting from the Phase 1 outcome. Three information-gathering pillars and a risk characterization pillar are situated across both phases. Each of these four pillars can be regarded as a toolbox containing data-generating tools (information-gathering pillars) or risk assessment tools (risk characterization pillar) that range from relatively simple to very complex.

The main goal of the Problem framing phase (Phase 1) is to identify Relevant Exposure Scenarios (RESs) for each step in the life cycle of an ENM and to check whether conclusions can already be drawn on the potential adverse health and/or ecological effects. Next, information and/or testing requirements, to be addressed in Phase 2, are defined, allowing for information compilation to be flexible and efficient. If possible, Phase 1 will provide a ranking or prioritization of the RESs according to the identified level of concern(s).

The Risk assessment phase (Phase 2) is a stepwise, iterative process of (a) identification of data gaps from the perspective of performing a risk assessment; (b) choosing the appropriate tools for data generation to fill in these gaps; (c) generation and collection of these data and (d) characterization of risks (*i.e.*, for human health and/or the environment, according to the user’s goals). An iterative process provides the opportunity to insert regular decision moments for the user to decide on the best option forward in information-gathering until the risk assessment goals of Phase 2 are met. These goals will be subjected to information requirements depending on the purpose of the user and the framework within which the assessment is performed.

The three information-gathering pillars represent the three basic domains of information: exposure, fate/kinetics and hazard. The risk characterization pillar contains tools for integration of the available information to perform a final characterization of risk(s) and to display the associated uncertainty. It is realized that information on physico-chemical properties is of great importance for ENMs and information on physico-chemical properties of the pristine material should be available from the beginning. This is important for substance characterization, as well as for exploring possibilities for grouping or read-across. Thus physico-chemical characteristics are part of the minimal base set of information for Phase 1 (see Section 2.2. Phase 1: Problem framing). It should be taken into account that the physico-chemical characteristics of an ENM may change over time and also that these characteristics may differ between RESs. It is of importance to identify these differences in order to collect and/or use information that is relevant for an ENM as it is present in each RES.

Further, physico-chemical characteristics of ENMs also provide information on exposure, fate/kinetics and hazard, as for example the affinity of a nanoparticle to the water or lipid phases would determine target organs. They can be used as input for models such as QSARs or for relatively simple exposure models that provide information on one or more of the domains of the information-gathering pillars. For that reason, these physico-chemical properties, and the methods to assess them, are considered to be an essential part of that domain (pillar) for which they provide insight or information and thus are included in the regarding pillar rather than to include them as a separate domain.

The strategy should be flexible such that the use of complex tools from one information-gathering pillar can be followed by a choice of relatively simple tools from another pillar in a later stage of the process or that relatively simple tools from one pillar can be used in combination with more complex tools from another pillar. Such flexibility is difficult to achieve by a strictly tiered approach in which the tools of all three domains become more complex with higher tiers and that is passed through in a one-direction process. Hence, the proposed risk assessment strategy in Phase 2 is a stepwise, iterative process that allows flexibility of choice of information-gathering tools.

The MARINA Risk Assessment Strategy triggers specific demands on the tools to be included in the respective pillars. The possibilities to include existing tools need to be verified, and the need to adapt existing tools or develop new ones should be explored. A separate paper on evaluation of the appropriateness of existing tools is in preparation [16]. The main elements will be described in more detail in the subsequent sections.

### 2.2. Phase 1: Problem Framing

The aim of Phase 1 in the MARINA Risk Assessment Strategy (orange disc in Figure 1) is to identify RESs throughout an ENM’s life cycle and to verify whether exposure in these scenarios may potentially lead to adverse health and/or ecological effects. As already mentioned, “relevant” in this context means relevant from the viewpoint of exposure, fate/kinetics and/or hazard and demanding further evaluation in Phase 2: Risk assessment. The main goal of the Problem framing phase is to set the scope for Phase 2 by defining information and/or testing requirements to be addressed and providing guidance for the strategy to collect this information in a flexible and efficient way. Exposure scenarios that are not considered relevant need no further elaboration; these scenarios should be documented including a rationale for further exclusion. The scope for Phase 2 may vary, depending on the goal of the user and its needs and/or the legal requirements of the framework in which the ENM is under evaluation. For instance, the strategy may be used during the design-phase of an ENM, for a risk assessment within a regulatory framework or for verifying possible risks in a specific exposure scenario of interest (e.g., with the highest exposure, regarding a specific application or a specific life-cycle stage).

Phase 1 is structured into two steps: (a) Data evaluation; and (b) RES identification. In order to properly identify RESs in Phase 1, there is a need to define an obligatory minimum base set of information requirements for an ENM, describing the physico-chemical properties and information for each of the three domains, *i.e.*, exposure, biological fate/kinetics and hazard. This minimum base set is defined such that, by combining the available information, RESs can be sufficiently determined, including potential relevant toxicological endpoints associated with each RES. The Phase 1 minimum information for human health includes e.g., information on local effects, absorption, potential for accumulation, genotoxicity, and immunotoxicity. For environment, this includes information on bioavailability, bioaccumulation/persistency and potential for toxicity for reproduction. It is noted that this minimum base set of information requirements as proposed in the MARINA Risk Assessment Strategy is not linked to any data requirements laid down in a specific regulatory framework, but it depends on the user’s goals and/or the regulatory framework in which the assessment is performed. These basic information requirements can be met by multiple options, including QSARs, Expert Systems, but also specific biological testing, if necessary. For example, if inhalation exposure is relevant for humans, evaluation of local effects on the respiratory tract is required in Phase 1 as well as tests for transfer across the lung epithelium (absorption) and potential for local and systemic accumulation. If the terrestrial environment is the most likely sink for ENMs, biological effect information should focus on this compartment, and link to possible human health exposure via environmental soil/dust. As the number of identified RESs increases across ENMs, it may be advantageous to create a library of relevant exposure scenarios. The three information-gathering pillars (see Section 2.4) should contain the tools and methods needed to generate the data on the three domains as required by the minimal base set.

Criteria need to be developed for making adequate decisions in Phase 1. It is noted that the basic information may already indicate in Phase 1 that an ENM may possess a too high risk potential to be allowed for a specific application or allowed at all (e.g., ENMs with an asbestos-like high aspect ratio). In practice, a risk assessor may then advise—If asked—Restraint. For industry the potential for high risk or negligible risk need to be taken into consideration in decisions on continuation of product or material development.

Further, the approach in Phase 1 and the information gathered can already be useful in the innovation chain during product or material development. The knowledge gained on the potential for health or environmental risks is relevant for decision making at many different levels, for example go/no go, safety-by-design, risk management measures, likelihood of regulatory acceptance, *etc.* These issues will be further addressed within the NANoREG project (http://nanoreg.eu).

#### 2.2.1. Step a: Data Evaluation

In the first step (step a) of Phase 1, the available data will be evaluated regarding completeness (in view of the minimum base set for the MARINA Risk Assessment Strategy) and quality, both aspects in relation to the use of the data, *i.e.*, for the identification of RESs and setting the scope for Phase 2. This ensures that Phase 2 starts only when sufficient and adequate information is available and RESs are identified.

It is expected that most often the ENM under evaluation will be a newly developed ENM, for which initially only basic information is available. In that case, the assessment of RESs will predominantly be based on physico-chemical properties, basic information on potential exposure (including e.g., production volume, emission, application, fate/kinetics and hazard from similar materials (if available), specific biological testing (according to the minimum base set of information requirements) and information on the expected life-cycle (from production to end of life). Additional information can for example be derived by use of simple tools, generic groupings, or basic release/exposure models and exposure libraries, continuously considering the uncertainty related to this information. In some cases, however, more detailed information may already be available and will then be taken into account in Phase 1. In general, all possible exposure scenarios throughout an ENM’s life-cycle, *i.e.*, from synthesis and formulation, via use to waste or disposal, need to be identified and grouped per route of exposure, e.g., respiratory, oral or dermal exposure for human health. Each route has unique characteristics that require specific information and tools for evaluation and thus demands specific guidance. For instance, extrinsic physico-chemical properties depend on the medium of exposure and thus may relate to the route of exposure.

It is recommended to consider possibilities for grouping already prior to entering Phase 1. For reasons of efficiency, it may then be considered to enter the strategy with a group of ENMs rather than with an individual ENM. The consideration of these possibilities within the MARINA Risk Assessment Strategy is subject of a separate paper [17]. This may improve the Problem framing since more information may become available but it also may enhance the efficiency if ENMs pass through the strategy as a group rather than as individual ENMs.

#### 2.2.2. Step b: RES Identification

In step (b) of Phase 1, RESs will be determined based on all available information. It is realized that especially for new ENMs, it will be impossible to foresee all possible future exposure scenarios (e.g., the possible applications/uses) but it is recommended to address as many scenarios as can reasonably be envisioned. When identified, risk assessment of these additional future scenarios may benefit from information gathered for RESs already addressed by the MARINA Risk Assessment Strategy. It is therefore recommended that the RESs that enter Phase 2 are chosen such that the information collected and the evaluation performed in Phase 2 will be representative for and/or applicable to multiple RESs.

First, the potential for exposure is analyzed. Important criteria to be considered for human exposure include likelihood of release of ENMs (and hence exposure) as well as the expected exposure concentration and/or dose and physico-chemical properties, the exposure frequency and duration and the population at risk. The RESs will be grouped according to the respective exposure routes as mentioned above, also considering the uncertainty in such estimates. The possibility that a population might be exposed via multiple routes will be addressed during the finalization process of Phase 2 (see Section 2.3.2 Finalization process). All available data will be evaluated and a flag will be assigned to each RES, either a green flag (no concern), a red flag (potential concern) or a black flag (insufficient information available) will be assigned. The concerns will be described based on the available information. To this end, it is expected that in time grouping and read-across will already be feasible for an increasing number of ENMs [17] which will provide the opportunity to enlarge the data set in Phase 1 thereby enabling better-founded evaluations.

For some ENMs (e.g., the ones with a green flag), Phase 1 may be the endpoint of the evaluation; for instance, if it can be concluded with a sufficient degree of certainty that risks are negligible. If, for example, exposure can be considered negligible with sufficient certainty (no significant contact between an ENM and an organism within all relevant exposure scenarios) a decision can be made in Phase 1 that no RES needing further evaluation has been identified. Some illustrative examples of possibilities for exposure-based waiving under the European REACH Regulation (EC No. 1907/2006) have been discussed by Vermeire *et al.* [18]. Another example is when the assessment concerns an ENM for which sufficient existing data are already available and therefore the conclusion on possible risks can already be drawn in Phase 1. On the other hand, it might also be possible that a too high risk is identified for an ENM, based on the information available in Phase 1, such that specific uses/applications are not allowable.

In most cases, Phase 1 will result in the identification and description of one or more RESs (including the most important issues to be addressed in Phase 2) and provide guidance for the collection of information in Phase 2. Also, different ENMs may enter Phase 2 as a group, if they share sufficient commonalities. This may for instance, be applicable to ENMs with a similar use and comparable physico-chemical properties, or in the case that the RES for one ENM is representative for other ENMs, or that the information to be collected in Phase 2 is applicable to multiple ENMs in RESs. In addition, if feasible, the RESs can be ranked per exposure route from high to low exposure potential with the scenarios showing the highest potential having the highest priority (priority ranking) for performing a risk assessment. The outcome of the Problem framing phase will be transferred to step (a) (Risk characterization) of Phase 2 (see Figure 1).

In case of a newly identified exposure scenario for a previously evaluated ENM, the first step will be to determine the similarities and differences between the new exposure scenario and the previously evaluated RESs to verify whether the information already available for the ENM is applicable to the new exposure scenario.

### 2.3. Phase 2: Risk Assessment

#### 2.3.1. Iterative Risk Assessment Process

The iterative Risk assessment process of Phase 2 consists of four steps (Figure 1, upper green disc):

Step (a)  *Risk characterization including risk management options*. This step focuses on whether the question on potential health and/or environmental risks can be sufficiently addressed. If yes, the risk can be characterized by using tools from the risk characterization pillar. If no, go to step (b) (Defining data needs).
Step (b)  *Defining data needs*. Identification of the data gap(s) with the highest priority to be addressed for the purpose of risk characterization, and definition of data or information that is required to optimally fill in this gap.
Step (c)  *Data gathering*. A compilation of data (including data generation) from one or more of the three information-gathering pillars, based on defined guidance, guidelines and other tools to ensure reliability and relevance.
Step (d)  *Data evaluation*. Evaluation of collected data in coherence with the data already available, including consideration of possibilities for read-across, grouping and data-sharing.

Step (a) *Risk characterization including risk management options.* In this step it is decided whether the available data are sufficient to finalize the risk assessment for a specific purpose defined in Phase 1. The available data should be adequate both in terms of quantity and quality to allow conclusions to be drawn with a sufficient level of certainty, and when this is the case, the risk assessment can be finalized. Four main conclusions are identified which could be drawn:

(i) The available data are insufficient to draw a final conclusion on risks with a sufficient level of certainty. The process proceeds to the next step of the evaluation cycle, *i.e.*, step (b) Defining data needs.
(ii) The available data are sufficient and negligible risks are expected. The iterative process ends and the results will be passed through to the finalization step (lower green disc in Figure 1, see below (Finalization process) for description).
(iii) The available data are sufficient and risks are present, but no adequate risk management options (RMOs), *i.e.*, identification of the best (regulatory) option to manage the risk, are available. The iterative process ends and the results will be passed through to the finalization step (lower green disc in Figure 1, see below (Finalization process) for description). It is not expected that gathering additional information would further refine the risk assessment. This conclusion may include that the exposure scenario considered should be avoided, which might imply that a specific application/use should be restricted or that the development of the ENM should be reconsidered.
(iv) The available data are sufficient and health/environmental risks are present and adequate RMOs are available. RMOs will be considered for their applicability and appropriate risk management measures (RMMs), if available, will be taken. The adequacy of the RMMs will be evaluated, and, if risks can be adequately controlled by RMMs taken, in principle no further evaluation is needed. It may, however, also be concluded that additional information is needed for the evaluation of the appropriateness of the RMMs, in which case another iteration of the evaluation cycle is needed.

Step (b) *Defining data needs*. In this step, the information is defined that best helps to address the identified information gap(s) and/or uncertainty for the purpose of risk assessment as well as how this information can be obtained, *i.e.*, which tools can be used. This information may either concern exposure, fate/kinetics and/or hazard and the choice is not limited to one tool but may include a set of tools or a battery of test systems from one or more information-gathering pillar(s) (see Section 2.4 Pillars). It should be realized that the information compilation may serve different purposes, e.g., to generate new information or to underpin grouping and read-across options, but also to verify the appropriateness of RMMs. The information may be obtained via different routes, for example, hazard or kinetic information may be obtained by actual testing. Exposure information may be obtained by using more sophisticated modelling or by performing actual exposure measurements. In other cases, the best option forward may be to obtain additional physico-chemical data (beyond the Phase 1 minimum base set requirements) with the aim to verify grouping or data-sharing possibilities for the ENM concerned, either on exposure, kinetics or hazard. If such a possibility can be applied, data gaps of the ENM can be filled in with relatively small efforts. Another example is collection of additional physico-chemical properties required for the application of more sophisticated models, such as physiologically-based pharmacokinetic modeling (PBPK-modelling).

In principle, the first choice of tools from the pillars are the relatively quick and simple tools, before proceeding to more complex and sophisticated (and more data-demanding) tools. One exception to this are clear cases that the available relatively simple tools will not sufficiently fill in a data gap, and then immediate use sophisticated tools may be the best option forward.

Step (c) *Data gathering*. The data or information defined in step (b) of the evaluation cycle is gathered and/or generated using the tools from the respective pillars as described in Section 2.4 (Pillars).

Step (d) *Data evaluation*. The new information is evaluated in coherence with the already available data assessing if and how the newly obtained information alters previously drawn conclusions in the domain (exposure, fate/kinetics, hazard) of the new information. This assessment is then passed through to step (a)) of Phase 2 to perform a risk characterization by using the tools included in the risk characterization pillar.

If, despite the new information or data, a risk characterization for a RES still cannot be appropriately performed, another iteration of evaluation is needed and more sophisticated tools can be used or information from another pillar can be obtained. The process is repeated until a risk characterization can be performed and/or additional data collection is not expected to further refine the risk assessment. The scope and level of detail of the final risk assessment and the associated acceptable uncertainty may depend on the user’s goal and/or the framework in which the risk assessment is performed. This will also determine the number of iterations necessary to meet the information requirements as defined in Phase 1.

#### 2.3.2. Finalization Process

In general, Phase 2 deals with one RES at a time (although the collected information may be applicable to multiple RES, see next paragraph) and is concluded by the finalization process (lower green disc in Figure 1). In this finalization process, the final outcome of the iterative risk assessment process will be summarized and the conclusions formulated in view of the goal of the user. This also includes the integration of effects in multiple tissues or compartments, combining data from multiple RESs and aggregation of data from multiple exposure routes. As described in the previous section, the iterative process ends when the available data are sufficient and no/negligible risks are expected (Conclusion (ii)) or when the available data are sufficient and risks are present, but no adequate RMOs are available (Conclusion (iii)); Conclusion (iii) in the risk characterization step may result in the conclusion that an intended use should be restricted or that the development of an ENM should be reconsidered.

As described above (in Section 2.2) it is recommended to define the scope in Phase 1 such that the efficiency of information gathering in Phase 2 is optimized, *i.e.*, that the information gathered will be applicable for multiple RESs whenever possible. Hence, once the risk assessment for a RES has been finalized it will be evaluated whether the conclusions drawn in the finalization step are partly or completely applicable to other RESs identified in Phase 1. If completely applicable, it will be evaluated whether these conclusions are sufficient to finalize the risk assessment also for these other RESs. If not or only partly applicable, the scope will be set for these RESs to enter the iterative information gathering and risk characterization process in Phase 2. The scope already established in Phase 1 will be adapted according to the additional information obtained for the RES that has been finalized.

In Phase 1, the RESs were grouped according to the exposure route. An exposure scenario may include multiple exposure routes for one population, e.g., both inhalation and dermal exposure during production or manufacturing. From an information-gathering point of view it appears reasonable to separate exposure routes for Phase 2 and consider them as separate RESs. However, applicability of information to other RESs (including exposure via multiple routes in one scenario) should be considered in step (b) (Defining data needs) during the iterative process, as mentioned in the previous paragraph. During the finalization process, the risks from multiple exposure routes within one exposure scenario can then be addressed.

When all identified RESs are appropriately addressed, an overall risk assessment for the ENM under evaluation is conducted. In this step, it will be evaluated whether specific populations might be exposed via multiple exposure scenarios during the life-cycle of an ENM. For instance, a worker exposed during the production of an ENM can also be exposed as consumer or consumers may come into contact via multiple applications of the ENM. An overall aggregated risk assessment will be performed to account for the potential of exposure to an ENM via multiple exposure scenarios. However, it should be taken into account that the ENM may have different extrinsic physico-chemical properties in the respective exposure scenarios which may complicate combining exposure and/or risk from different scenarios.

### 2.4. Pillars

The three information-gathering pillars which are cross-linked with both phases form another main element of the MARINA Risk Assessment Strategy. These three pillars are considered as toolboxes, containing a number of tools to generate the required data. They are part of the Data evaluation step (step (a)) in Phase 1 and of the Data gathering step (step (c)) in Phase 2. An exposure scenario describes how different (human and/or ecosystems) populations are exposed to an ENM, e.g., the route of exposure, duration, frequency and level. To be able to assess risks in a defined exposure scenario, the information on environmental fate/kinetics and hazard to be obtained needs to be tailored to that scenario. Tools are then needed in Phase 1 to translate uses and applications into a first exposure estimate, but also to translate physico-chemical characteristics into potential toxicity endpoints, e.g., via QSARs, Expert Systems or Data-sharing systems. Once an exposure scenario is defined, information on biological kinetics and fate comes into focus. Kinetic or fate information is needed to describe what happens to an ENM once it comes into contact with the environmental or a biological system and to translate an external exposure estimate into a relevant biological dose descriptor, and to determine the potential for accumulation, persistency and toxicity. Fate/kinetics help to identify potential area/target sites (e.g., organisms, organs/tissues) and the relevant biological exposure levels to be addressed in hazard testing. Hazard information may include the endpoints of (potential) concern, mode of action or description of the toxicity pathway, and dose-response information. These three pillars are therefore closely linked, and should not be treated as independent domains but be considered as integrated toolboxes in all steps.

The tools in the risk characterization pillar are not meant to generate new data themselves but are used as methods to optimally interpret, evaluate and integrate the available information. This pillar is part of the RES identification step (step (b)) in Phase 1 and of the Risk characterization step (step (a)) in Phase 2. In Phase 1, these tools are to be applied primarily for the purpose of RES identification, selection and prioritization. Examples may be the Threshold of Toxicological Concern (TTC) approach (not yet specified for ENMs) or specific ranking tools. In Phase 2 the focus is more on quantitative approaches and more sophisticated tools are available; the risk characterization pillar is included in step (a) (Risk characterization).

**Figure 2:**
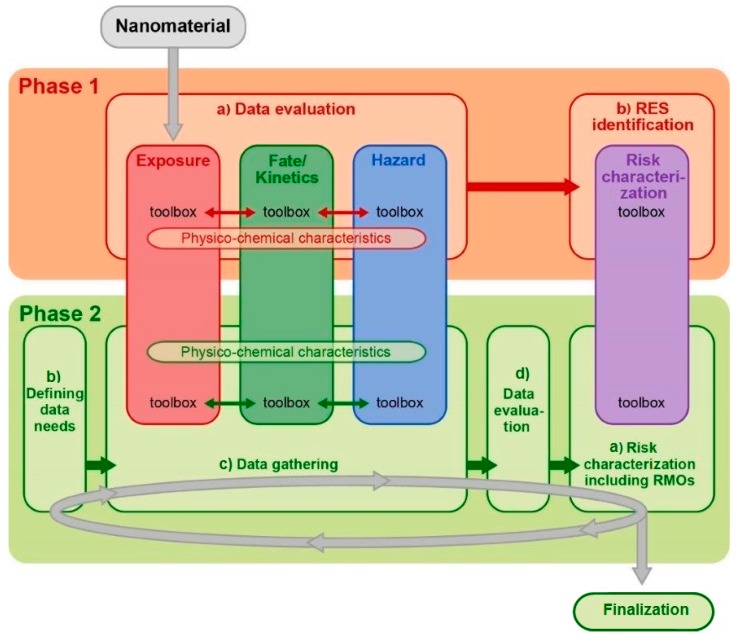
Schematic view of the three information-gathering pillars on the left and the risk characterization pillar on the right and their placing in the respective phases and steps of the MARINA Risk Assessment Strategy.

Each pillar contains a number of tools serving different purposes and ranging from relatively simple to complex tools. Their placing in the MARINA Risk Assessment Strategy is depicted in Figure 2. As already indicated, the pillars should not be considered as separate units, but knowledge from one pillar may, for instance, be needed to either apply a tool from another pillar or steer the application of a tool in another pillar. Examples of the former include the necessity that a quantitative hazard estimate is to be assessed before a quantitative exposure estimation (e.g., by actual measurements) might be considered meaningful or that fate/kinetic information on the potential to reach the fetus or embryo can be considered obligatory before considering to study developmental toxicity. Further, output from one tool may become input for another tool of the same or of another pillar which put specific demands on the development of tools. For instance, tools from the risk characterization pillar need to be able to use the output of tools from the three information-gathering pillars.

The complexity of the tools chosen from the respective pillars will, in general, increase as the risk assessment process moves from Phase 1 to Phase 2, and with each iteration performed. The tools should support risk screening and prioritization in Phase 1 and risk assessment in Phase 2, with, in general, more qualitative tools for Phase 1 purposes and more quantitative (probabilistic) tools (including uncertainties) for Phase 2 goals. However, general tools for uncertainty estimates should be applicable throughout; uncertainty propagates through the whole risk assessment process. Uncertainty can be associated with variability or insufficient knowledge about relevant mechanisms of toxicity or exposure scenarios, as well as with the model implementation and its variables. A description of available and under development tools and methods that could be used in the MARINA Risk Assessment Strategy is the subject of a separate paper [16].

## 3. Discussion

The risk assessment of ENMs has specific needs and makes specific demands that need to be addressed in careful detail, and the current risk assessment methodologies and frameworks do not provide sufficient guidance for this. In addition, the combination of a wide variety of ENMs and possible changes during the lifecycle of one EMN means that the sheer number of ENMs to be assessed requires a new approach. The challenge is to develop an efficient risk assessment strategy with the precondition that the strategy ensures the safety of ENMs as such and in products. Efficiency is needed because ENMs can vary in multiple physico-chemical properties that affect exposure, fate/kinetics and/or (eco)toxicity and thus can affect risk. Furthermore, ENMs of a particular chemical composition may actually consist of a varying population of primary particles, aggregates and agglomerates of various sizes, and different surface coatings. Testing of each slightly different ENM and each member of such a population will be very resource intensive, and other ways of assessing possible risks are sought. For that reason, the MARINA project investigated the possibilities for a risk assessment strategy that considers the impact of the varying properties of an ENM during the various life cycle stages on human health and environmental risks and at the same time requires minimum data.

The concept for a risk assessment strategy for ENMs is presented. The main advantage of this strategy is the iterative approach that allows a high level of flexibility to generate only data needed for a user’s purpose. The strategy is exposure-driven, *i.e.*, focusing on defining relevant and realistic exposure scenarios, which may go beyond standard exposure scenarios; within these scenarios possible hazard endpoints are considered. Setting up a RES library would be one way of consolidating the accumulated experience. These RESs are identified in Phase 1: Problem framing that sets the scope for defining the information requirements for Phase 2: Risk assessment. Another important aspect of the strategy is the integration of exposure, fate/kinetics and hazard throughout the whole process, especially during data-gathering and for the risk characterization. Determination of physico-chemical properties of an ENM and their meaning for exposure, fate/kinetics and hazard is integrated in the strategy, as well as analytical quality and suitability of tools and methods for an ENM or RES. The final scope and level of detail of the risk assessment and the associated uncertainty may depend on the user’s goal and/or defined by the framework within which the risk assessment is performed. These goals may include a full risk assessment within a regulatory context but also targeted testing in an ENM design-phase or risk assessment for a specific exposure scenario such as a specific application of an ENM.

The presented strategy, including its basic principles, and the underlying components are in line with the recommendations and needs as described by the ITS-NANO project [15]. The ITS-NANO project identified short-, mid-, long-term and distant future research priorities to develop approaches that would allow the development of an Intelligent Testing Strategy for ENMs. The concept framework of the MARINA Risk Assessment Strategy provides an outline in which these approaches can be practically implemented in accordance with the ITS-NANO recommendations. It also meets a range of expectations as presented by the EU Scientific Committees in 2013 [10], such as starting from realistic exposure scenarios, the use of grouping and read-across and computational tools in an early stage of the risk assessment process and on the integration of kinetic data.

An important issue that remains to be solved is the identification of decision moments in Phase 1 and in the iterative process of Phase 2. Development of guidance for these decision moments is required, including decision criteria and the assessment of associated uncertainty. For instance, in Phase 1 guidance needs to be provided on (i) how to define the individual ENM or group of ENMs to be assessed, (ii) how to identify, select and prioritize RESs, depending on the goals of the user and on (iii) how to proceed from Phase 1 to Phase 2. This also includes the identification of possibilities for a combined evaluation of RESs in Phase 2. Further, a(n) (obligatory) minimal dataset needs to be determined for Phase 1. In Phase 2, guidance is needed on how to obtain the identified relevant information and on how to meet the requirements in terms of data quantity and in quality (e.g., test guideline to be followed, quality control of already existing information). Another issue concerns how to assess uncertainty qualitatively and/or quantitatively. In addition, guidance is needed on how to define the most important data and on the choice of appropriate tools or sets of tools in step b) of Phase 2. This will be subject of future research in other collaboration projects on risk assessment of ENMs. Regular performance of case studies will be needed to verify the feasibility and practicability of the strategy and to identify where adjustment is needed. Possibilities for grouping and read-across within the strategy, and a review of tools and methods that could be applied in the MARINA Risk Assessment Strategy for ENMs are subject of separate papers prepared within MARINA.

## 4. Conclusions

In conclusion, the concept of the MARINA Risk Assessment Strategy has been developed to meet the challenges involved in efficient information-gathering for risk assessment of ENMs and in the risk assessment process itself. The basic process of this exposure-driven strategy consists of two phases, *i.e.*, a Problem framing phase and an iterative Risk assessment phase. Further developments are needed to make the strategy operational. For example, there is a need to provide essential guidance and tools, for information-gathering and risk characterization as well as for the decision-making moments.

## References

[1] Mitrano D.M., Motellier S., Clavaguera S., Nowack B. (2015). Review of nanomaterial aging and transformations through the life cycle of nano-enhanced products. Environ. Int..

[2] Arts J.H., Hadi M., Keene A.M., Kreiling R., Lyon D., Maier M., Michel K., Petry T., Sauer U.G., Warheit D. (2014). A critical appraisal of existing concepts for the grouping of nanomaterials. Regul. Toxicol. Pharmacol..

[3] Arts J.H., Hadi M., Irfan M.A., Keene A.M., Kreiling R., Lyon D., Maier M., Michel K., Petry T., Sauer U.G. (2015). A decision-making framework for the grouping and testing of nanomaterials (DF4nanogrouping). Regul. Toxicol. Pharmacol..

[4] Oomen A.G., Bos P.M., Fernandes T.F., Hund-Rinke K., Boraschi D., Byrne H.J., Aschberger K., Gottardo S., von der Kammer F., Kuhnel D. (2014). Concern-driven integrated approaches to nanomaterial testing and assessment—Report of the nanosafety cluster working group 10. Nanotoxicology.

[5] Landsiedel R., Fabian E., Ma-Hock L., van Ravenzwaay B., Wohlleben W., Wiench K., Oesch F. (2012). Toxico-/biokinetics of nanomaterials. Arch. Toxicol..

[6] Pietroiusti A., Campagnolo L., Fadeel B. (2013). Interactions of engineered nanoparticles with organs protected by internal biological barriers. Small.

[7] National Research Council (2007). Toxicity Testing in the 21st Century—A Vision and a Strategy.

[8] Krewski D., Andersen M.E., Mantus E., Zeise L. (2009). Toxicity testing in the 21st century: Implications for human health risk assessment. Risk Anal..

[9] Andersen M.E., Krewski D. (2009). Toxicity testing in the 21st century: Bringing the vision to life. Toxicol. Sci..

[10] European Union (2013). Addressing the New Challenges for Risk Assessment.

[11] Tantra R., Oksel C., Puzyn T., Wang J., Robinson K.N., Wang X.Z., Ma C.Y., Wilkins T. (2015). Nano(Q)SAR: Challenges, pitfalls and perspectives. Nanotoxicology.

[12] Scientific Committee on Emerging and Newly-Identified Health Risks (2007). The Appropriateness of the Risk Assessment Methodology in Accordance with the Technical Guidance Documents for New and Existing Substances for Assessing the Risks of Nanomaterials.

[13] Organisation for Economic Co-operation and Development (2012). Important Issues on Risk Assessment of Manufactured Nanomaterials.

[14] Organisation for Economic Co-operation and Development Guidance on Sample Preparation and Dosimetry for the Safety Testing of Manufactured Nanomaterials. Http://www.Oecd.Org/officialdocuments/publicdisplaydocumentpdf/?Cote=env/jm/mono%282012%2940&doclanguage=en.

[15] Stone V., Pozzi-Mucelli S., Tran L., Aschberger K., Sabella S., Vogel U., Poland C., Balharry D., Fernandes T., Gottardo S. (2014). ITS-NANO—Prioritising nanosafety research to develop a stakeholder driven intelligent testing strategy. Part. Fibre Toxicol..

[16] Hristozov D., Gottardo S., Semenzin E., Oomen A., Bos P., Peijnenburg W., van Tongeren M., Nowack B., Hunt N., Brunelli A. Frameworks and tools for risk assessment and management of manufactured nanomaterials. Environ. Int..

[17] Oomen A.G., Bleeker E., Bos P., van Broekhuizen F., Gottardo S., Groenewold M., Hristozov D., Hund-Rinke K., Irfan M.-A., Marcomini A. (2015). Grouping and read-across approaches for risk assessment of nanomaterials. Int. J. Environ. Res. Public Health.

[18] Vermeire T., van de Bovenkamp M., de Bruin Y.B., Delmaar C., van Engelen J., Escher S., Marquart H., Meijster T. (2010). Exposure-based waiving under reach. Regul. Toxicol. Pharmacol..

